# Teaching early-career researchers how to respond to peer reviewers

**DOI:** 10.7554/eLife.102619

**Published:** 2026-04-23

**Authors:** Eric Kalkhoven, Manon Kluijtmans

**Affiliations:** 1 Center for Molecular Medicine, University Medical Center Utrecht, Utrecht University Utrecht Netherlands; 2 Center for Education, University Medical Center Utrecht, Utrecht University Utrecht Netherlands; 3 University College Utrecht, Utrecht University Utrecht Netherlands

**Keywords:** Point of View, early-career researchers, peer review, scientific publishing, None

## Abstract

The process of publishing a research article in a scientific journal inevitably involves revising the original version of the article to respond to the concerns raised by peer reviewers. In this article we describe a course module that introduces MSc students at Utrecht University in the Netherlands to this part of the publication process. During the module the students and an invited speaker actively discuss the revision process for a recent article by the speaker. Feedback from students and speakers on the module – which could be readily transferred to other courses in the life and biomedical sciences – has been largely positive.

## Introduction

Early-career researchers in the life and biomedical sciences have to publish articles in peer-reviewed research journals in order to advance in their career. This is why many postgraduate degree courses include modules on writing scientific articles. However, writing an article and submitting it to a journal is just the first stage in the publication process. If the journal decides to review the article it will be sent to a small number of experts in the field, and these ‘peer reviewers’ will write reports on the strengths and weaknesses of the article (Rennie, 2016; Kelly et al., 2014; Thistlethwaite, 2012).

If these reports are mostly negative, the article will probably be rejected, and the authors will have to submit it to a different journal. If, however, the reports are more positive, it is likely that the authors will be asked to revise their article and resubmit it, along with a point-by-point response or rebuttal to all the points made by the peer reviewers. For each of these points this rebuttal generally includes the original point from the reviewer, a response to that point from the authors, and a summary of how the article has been revised in response to that point. Since journals typically use 2–4 reviewers per article, and each reviewer can make multiple points, these rebuttals can run to 20 or more pages.

How the authors respond to the points made by the reviewers has a strong influence on whether or not the revised article will be accepted for publication (Kelly et al., 2014; Bourne, 2005; Nature Geoscience, 2019; Samet, 1999; Woolley, 2018). However, to the best of our knowledge, early-career researchers are rarely taught about this aspect of the publication process (McDowell et al., 2019).

This article describes a course module that is used to introduce master’s students at Utrecht University in the Netherlands to this crucial part of the research cycle. The module centers around interactions between the students and an invited speaker, including a discussion of the revision process for a recent article by the speaker.

## How the module works

The module – which has been running since 2018 – is part of course on gene expression, epigenetics and disease, so the invited speaker is always an active researcher in one of these areas. The students are expected to spend five hours on the module, including preparation, and there is a different invited speaker every year. Following the module, the invited speaker gives an institute-wide seminar, which the students also attend.

**Figure 1.**
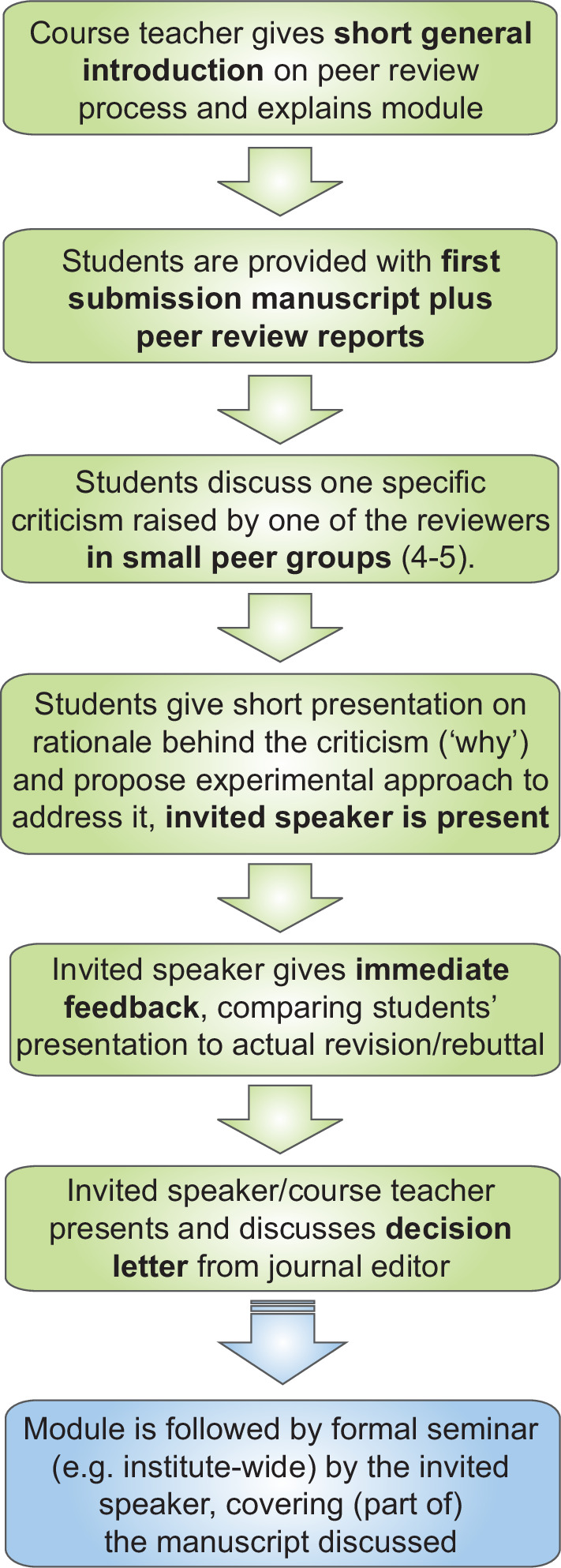
A module on the revision and rebuttal process in peer review. After a short general introduction on the review process, the module starts with students reading the original version of a published research article, along with the reviewer reports on this version. The students then form small groups to discuss one comment made by one of the reviewers. (Each group addresses a different comment). The students then give short presentations on the reasons for the comment, and outline how they would address it. The author of the paper (the invited speaker) gives feedback on the presentations, and explains how they responded to the comment. Finally, there is a discussion of the decision letter from the editor. Following the module, the invited speaker gives an institute-wide research seminar, which the students also attend.

After a short general introduction on the peer review process, each session starts with the students breaking into small groups (4–5) to study the manuscript and the points and comments made by the reviewers (Figure 1). Subsequently, each group focuses on a specific comment: what is the rationale behind the comment (‘why’), and what would be an appropriate experimental approach or analysis to address it (‘how’). With this ‘how’ question, the module deepens the students’ subject knowledge and stimulates creative thinking in a scientific context, as well as enhancing their understanding of scientific practice.

Each group then gives a short presentation (10 minutes; 2–3 slides; see Supplementary file 1 for an example) on the ‘why’ and the ‘how’ for the specific comment they discussed, and questions are asked by fellow students. The invited speaker also provides immediate feedback on whether the students have understood the reviewer’s point and interpreted it like the speaker and their team did, and whether the experimental approach being proposed by the students is appropriate. The speaker also reveals how he/she has responded to the reviewer ‘in real life’.

The session concludes by discussing the decision letter from the editor of the journal, which may contain hints as to which extra experiments (and other revisions) the editor deems to be essential, and which might be optional, assuming the authors can provide a compelling rationale for not performing certain extra experiments.

To illustrate how the module works in practice, we present actual examples based on four articles discussed by the invited speakers. The examples cover four different scenarios: in some of these the authors have performed extra experiments or made various changes requested by the reviewers, but in others they have pushed back and explained why the requested experiments/changes were not necessary. In each of these examples, peer review material is available for the article on the journal website.

### Authors perform extra experiments

Marieke Oudelaar of the Max Planck Institute for Multidisciplinary Sciences in Göttingen spoke about experiments in which they reconstituted yeast (*S. cerevisiae*) in vitro to study 3D genome organization, and showed that the spacing of nucleosomes alone can organize DNA into 3D structural domains (Oberbeckmann et al., 2024). Partially building on in vivo data reported by others, their studies implicated both transcription factors (TFs) like Abf1 and Reb1, as well as chromatin remodelers, in setting genome domain boundaries (a process called insulation) in vitro.

One group of students was asked to consider the following comment from reviewer #3: *“It has been previously shown that binding of multiple transcription factors can drive insulation. The authors make the assumption that Abf1 and Reb1 bind in vitro based on the in vivo ChIP- seq data, but the manuscript does not directly demonstrate that Abf1 and Reb1 TFs are actually able to bind DNA in the in vitro conditions chosen, in the absence of chromatin remodelers.”* The referee went on to state that certain ChIP-seq experiments were necessary to address their concerns, but this information was not shared with the students.

The students agreed that the article needed to contain evidence to show that Abf1 and Reb1 could indeed bind to DNA in the in vitro system used. Therefore, to address the comment from the reviewer, they proposed a number of ChIP-seq experiments (see Supplementary file 1 for details). This response turned out to be similar to what the authors did (and what the reviewer had suggested).

The authors responded as follows: *“We would like to thank the Reviewer for these helpful suggestions. We had not yet performed in vitro ChIP-seq experiments, since the appearance of nucleosome-free regions at binding sites for Abf1 and Reb1 suggests that these transcription factors bind to DNA in the in vitro conditions in presence of remodelers. However, we agree with the Reviewer that it is important to confirm the binding of the transcription factors in vitro in presence of remodelers and to test if the transcription factors also bind in absence of the remodelers. We therefore performed ChIP-seq experiments for both Abf1 and Reb1 in all experimental in vitro conditions. The resulting data are shown in Extended Data Figure 2, which we have copied below. These data clearly show that Abf1 and Reb1 bind to DNA, both in presence and in absence of chromatin remodelers.”*

### Authors decline to perform extra experiments

Denis Duboule of the EPFL in Lausanne spoke about an article in which he and his colleagues found that multiple DNA regulatory elements work together to control the development of external genitals in mice. They also found that many of these regulatory elements can be removed without strongly affecting gene activity. Their analyses zoomed in on a key developmental gene called Hoxd13, and one of the regulatory sub-elements they studied was called GT1. Overall, they concluded that gene regulation in this region is robust and buffered by redundant control elements (Amândio et al., 2020).

One group of students was asked to consider the following comment from one of the reviewers: *“It would be of great interest to see: would the precise deletion/mutation of GT1 also lead to upregulation of Hoxd13.”*

The students appreciated how addressing this point may strengthen the manuscript and provided an elaborate in vivo approach to address the question; nonetheless, when presenting their ideas, they expressed some doubts as to whether investigating the GT1 sub-element in detail would be worthwhile.

In their response to the reviewers, the authors explained why they had decided not to do extra experimental work to address this point. *“We certainly understand this interest for the GT1 sequence. We nevertheless decided to focus this work on the central and telomeric parts of C-DOM, rather than on the centromeric part containing GT1, mostly because the central deletion (and that of Prox) had the strongest impact on Hoxd13 transcription […] While of potential interest, this investigation would require years of work using embryonic material and is in fact not on the agenda”*.

### Authors perform extra experiments, and ‘soften’ language in article

James Lee of the Francis Crick Institute in London spoke about an article in which he and his colleagues linked a small DNA element called an enhancer, located far from any genes in the genome, to several inflammatory diseases. They reported that genetic variation in this enhancer increased expression of a transcription factor called ETS2 in immune cells, thereby driving inflammation. Their findings indicated a shared mechanism for the inflammatory diseases, and thus suggested new targets for anti-inflammatory therapies. The authors also speculated that the enhancer variant may result in a feed-forward loop, where enhanced ETS2 expression increases the activity of the enhancer, thereby increasing the expression of ETS2 even further (Stankey et al., 2024).

One group of students was asked to consider the following comment from reviewer #1: *“Lines 296: The findings concerning binding of ETS2 to its own enhancer is of importance and interest, but the suggested feed-forward loop is speculative”*.

The students suggested two experimental approaches that could be used to address this comment: one involved genetic disruption of the ETS2 binding site, and the other involved overexpression of ETS2.

The authors agreed with the reviewer that this part of the article was speculative, and performed a number of experiments to address the comments. These experiments and the experiments suggested by the students were remarkably similar. Here is what the authors wrote in their response: *“Thank you for this suggestion. We agree that our wording may have been too speculative and have now performed two complementary experiments to test the positive feedback loop that we proposed…. [Authors describe experiments in detail]….The results are therefore consistent with ETS2 contributing to the activity of its own enhancer, but we cannot exclude a contribution from the associated changes in inflammation. We have therefore softened the language used in the main text and have added the supporting data to Extended Data Fig.6g-i”*.

### Authors use preliminary data to explain lack of revision

Judith Zaugg of the EMBL in Heidelberg spoke about an article in which she and her colleagues used a machine learning approach to show that certain DNA features – especially in gene promoters – can predict how much gene activity varies between individuals, and how genes respond to changes. This indicates that built-in genomic features of genes partly determine their responsiveness to environmental or biological perturbations (Sigalova et al., 2020). Their data was based on cell populations (‘bulk data’), and would not address cell-to-cell variation.

One group of students was asked to consider the following comment from reviewer #3: *“Are the same genomic features predictive of transcriptional precision among individuals also applicable to single cells within a tissue or cell population?”*.

The students discussed this comment, and proposed a bioinformatics approach to address it.

The authors generated preliminary data in an effort to provide support for the connection suggested by the reviewer, but in the end they decided not to include these data in the revised manuscript. This is what they wrote in their response: *“This is an interesting question. We expect yes, as our study using bulk data has uncovered many properties that were identified in individual single cell studies, such as a TATA box, Pol II pausing etc. We agree with the reviewer that it would be interesting to address this directly. Please note that direct comparisons of bulk and single cell data are complicated by the differences in composition of expression variation (e.g. intrinsic and extrinsic noise)….[Authors nevertheless provide preliminary data to the reviewer]…. Despite these encouraging results, we would prefer not to add them to our manuscript since these are very preliminary and will require further confirmation on a larger number of studies, and in-depth analysis of single-cell expression noise is non-trivial. We therefore believe it is out of scope for this manuscript.”*

### The decision letter from the editor

In addition to the reviewer reports, the students and the invited speakers also discussed decision letters from editors. These letters often provide guidance on which comments from the reviewers must be addressed in order for a revised version of the article to be accepted for publication.

The decision letter sent to James Lee and colleagues about their paper on ETS2 (Stankey et al., 2024), for example, makes clear that a revised version is unlikely to be accepted unless the authors can show that ETS influences more than one inflammatory disease:

“Your manuscript, A disease-associated gene desert orchestrates macrophage inflammatory responses via ETS2, has now been seen by 4 referees, whose comments are attached below. While they find your work of potential interest, as do we, they have raised important concerns that in our view need to be addressed before we can consider publication in Nature. Should further experiments allow you to address these criticisms, we would be happy to consider a revised manuscript …. There are a fairly substantial number of issues but I suspect the most challenging practically-speaking will be to demonstrate that the effects of ETS2 are generalizable to other diseases beyond IBD [inflammatory bowl disease]. Addressing this would broaden the appeal and I suspect greatly increase the chances of endorsement by these referees.”

Lee explained how he and his co-authors authors thoroughly revised their manuscript, addressing many of the criticisms (experimentally or otherwise, along the lines outlined above) and, most importantly, broadening their findings to other diseases beyond IBD. The revised manuscript was then accepted for publication.

## Feedback from students and invited speakers

Student experiences were quantitatively and qualitatively evaluated in the first three editions of the module (see Supplementary file 1). To triangulate the data on student perceptions, the invited scientists were also asked to reflect on their experiences.

### Feedback from students

Students were positive about the module, giving it an average rating of 4.5 out of 5 for being “helpful and instructive” (Likert scale from 1 (strongly disagree) to 5 (strongly agree); n=111; response rate 96%).

Thematic analysis of written feedback (n=82) provided insights into three themes: (i) affective experience (i.e. emotional reactions during the task performance); (ii) understanding of scientific practice; (iii) understanding of course content.

With respect to the first theme, affective experience, the students mostly found the experience enjoyable and useful, and indicated that it was unique within the curriculum, as illustrated by these quotes: “This was great. I hope that students following the course in the next year will also have the opportunity to do it”; “Very cool and helpful assignment! Have never had something like this in a course”.

In addition, the interaction with the corresponding author and the ‘real life’ aspect of the module were clearly valued: “The assignment was interesting to do. I liked very much the discussion with the author and the explanation how he addressed the comments”; “I really liked this as you look at the results from a different perspective & direct feedback from the author”.

However, some students were less positive: “Not really clear to me”; “It was very challenging”.

With respect to the second theme, understanding of scientific practice, the comments showed that the module had introduced many of them to the revision and rebuttal process for the first time. Some also commented that the module had helped to prepare them for a future job in research: “It offered a nice view of what reviewers look for in a paper and it was an occasion to compare how students would solve a problem and how a researcher does it. It was a great opportunity for discussion”; “I really liked this exercise, very useful as part of being trained as a scientist”.

In terms of the Dreyfus model of skills acquisition, we can say that the module lets the students move from ‘novice’ (stage 1) to ‘competent’ (stage 2) regarding their skills in the revision and rebuttal process (Dreyfus and Dreyfus, 1980). Competent means that they will be able to recognize similar situations or settings, and to apply guidelines: however, they still need to acquire ‘proficiency’ (stage 3) and ‘expertise’ (stage 4) to reach ‘mastery’ (stage 5). One way for a student to move from stage 2 to stage 3 and beyond is to co-review an article with an established researcher (McDowell et al., 2019; Macarelli and Merkle, 2025). When asked to review a manuscript, many senior researchers already do this, and many journals encourage reviewers to involve a junior member of their group in the review process (Nature, 2025; Gierasch, 2019).

The third theme, understanding of course content, emerged as a result of the students being asked to analyze a complex manuscript in detail and to design new experiments: “Really liked the concept. Never done before. It lets you think and practice how to solve questions by performing experiments”; “It was super cool and gave opportunities to design experimental approaches”.

While the feedback from students was mostly positive, a few found the module challenging or unclear in purpose, which may be interpreted as them being outside their comfort zone. In social-constructivist learning theory this is referred to as the ‘zone of proximal development’, and being outside this zone can support learning, even if it feels uncomfortable to students (Vygotsky and Cole, 1978).

### Feedback from invited speakers

Three of the invited speakers also provided comments, which were all positive. Stefan Mundlos of the Max Planck institute for Molecular Genetics in Berlin said: “I very much enjoyed the course and think it is a great idea! The discussion with the students often brings in new aspects that no one else has thought about.”

Michiel Vermeulen of the Radboud Institute of Molecular Life Sciences in Nijmegen said he “thoroughly enjoyed” the exercise and his interaction with the students: “It was interesting to see them act as a reviewer of my own research paper. Furthermore, the discussions with the students also touched upon the various issues that need to be considered when dealing with editors from journals to get your work published. The subtleties of reading between the lines, interpreting an editorial decision letter, etc. I could sense that the students really enjoyed becoming familiar with this important part of doing science”.

Denis Duboule of EPFL said he was “initially surprised” by the request to participate: “However, I rapidly realized how useful it could be both for the students and for myself. The meeting in Utrecht was even better than anticipated, with a detailed knowledge of the publication under scrutiny and many good ideas on what should be done in a revised version. A very nice way to introduce students into the real life of a scientist, with all the fun and the frustration. Great experience.”

## Conclusions

We designed and evaluated a module on the revision and rebuttal process in peer review for master’s students. The key principles of the module – which could readily be implemented in other courses – are as follows: (i) it is based on a real-life examples of the revision and rebuttal process; (ii) the students critically evaluate the points made by reviewers with their peers; (iii) the students interact with an invited speaker of international renown, who gives immediate feedback on their responses; (iv) the invited speaker openly shares their experiences of the peer review process with the students, including the art of interpreting comments from the reviewers and the editor, and how to respond to these comments. The module has been well received by the students and the invited speakers. Embedding the module in a discipline-specific course also deepens the students’ understanding of the course content.

Ultimately, we feel that this type of real-life training helps students to ‘think, act and feel’ as researchers, and to develop their professional identity (Merton et al., 1957; Cruess et al., 2019). Moreover, we believe that the module described in this article could be a valuable addition to MSc and PhD programs across the life and biomedical sciences.

## Data Availability

There are no experimental data associated with this work.
