# Supplementary material for: Teaching early-career researchers how to respond to peer reviewers
Source: eLife. 2026 Apr 23;15:e102619. doi: 10.7554/eLife.102619 (PMC13105477; doi:10.7554/eLife.102619)

Kalkhoven E, Kluijtmans M. 2026. Point of View: Teaching early-career researchers how to respond to peer reviewers. *eLife* **15**:e102619. https://doi.org/10.7554/eLife.102619

**SUPPLEMENTARY FILE 1**

**Example of student presentation**

**Methods: Setting of the module and evaluation**

**Methods: Data analyses**

**Table: Students’ learning experiences: main themes and subthemes**

**Figure: Distribution of quantitative students’ scores**

**Example of student presentation**

Slides presented by students about a reviewer comment on Oberbeckman et al. (*Nature Genetics* **56**:483–492; 2024), and their suggestion for addressing this comment.


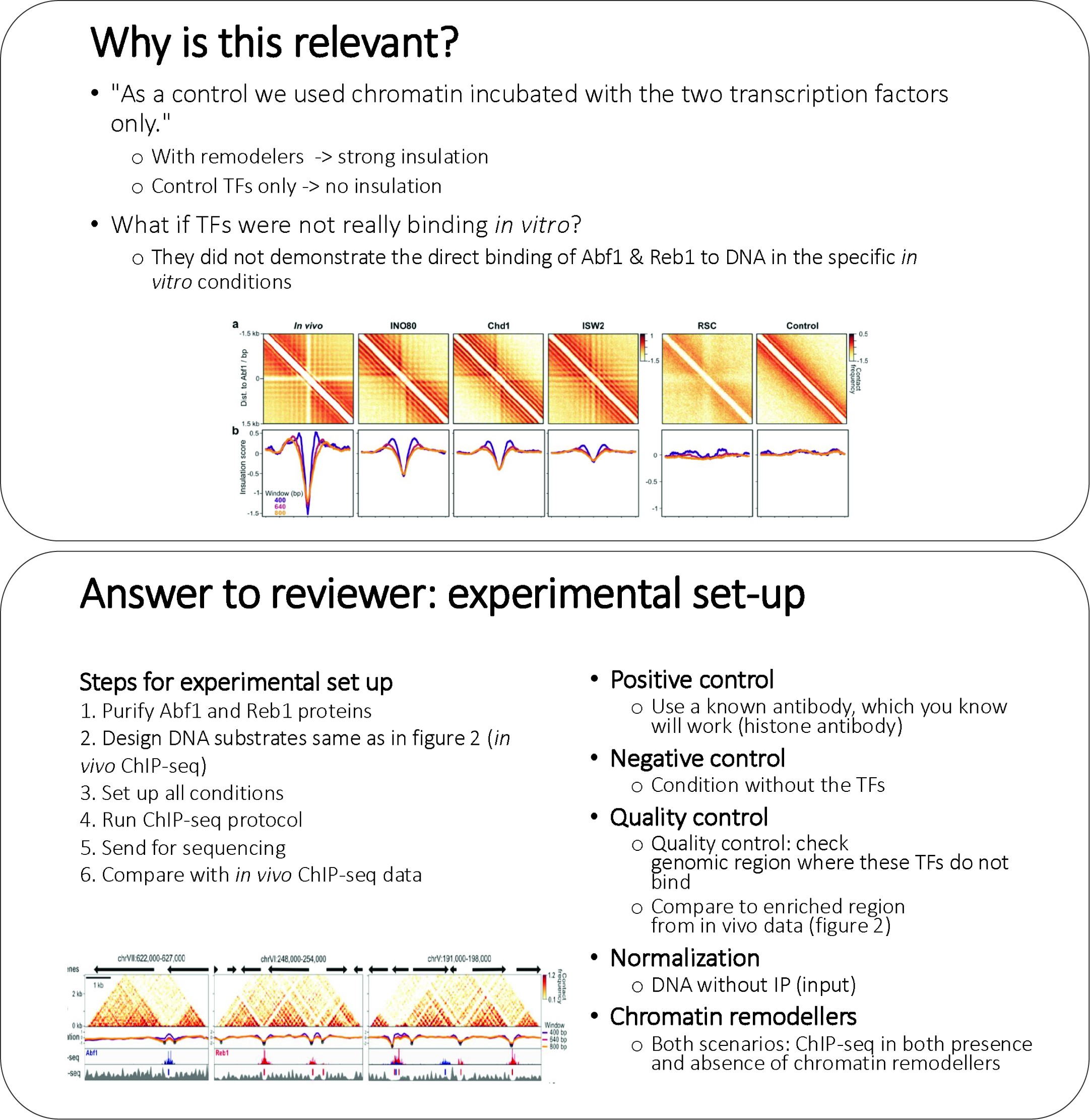


**Methods: Setting of the module and evaluation**

The module consisted of ∼5 hours (including preparation), and was integrated into an existing MSc course named “Gene expression, epigenetics and disease”, a 3 EC full time elective course, which is part of the MSc in BioMedical Sciences master program “Cancer, Stem cells and Developmental biology” (CS&D; <https://www.csnd.nl/>) at the Utrecht Graduate School of Life Sciences (GSLS; <https://www.uu.nl/en/education/graduate-school-of-life-sciences>), Utrecht University, the Netherlands. The first author, EK is one of the coordinators of this course and was the initiator of the new module. The module was first introduced in 2018 and has been running yearly since.

To gain insight into the learning experiences of students an exploratory evaluation was conducted on the evaluation data of the first three cycles of the course: 2018-2020. Immediately after the course, all participants of the first three editions of the course containing the new module were invited to complete a specific evaluation form in English on the rebuttal module in the classroom, next to a standard anonymous questionnaire on the course as a whole. This evaluation was part of standard progam evaluation and therefore the study was exempt from ethical review. Hand-written evaluation results were collected anonymously. Participation was completely voluntary and had no influence on their study program.

An exploratory analysis of these evaluation data was conducted to obtain insight into student satisfaction and learning gains over 3 subsequent editions. As a quantitative measure, students were asked to score whether the module was “helpful and instructive” on a Likert scale from 1 (strongly disagree) to 5 (strongly agree). To gather qualitative data on why the students gave the respective scores, ample room for written feedback was provided.

**Methods: Data analyses**

Quantitative data were collected and are represented for each edition and collectively (see Figure). The qualitative data on students’ learning experiences and the researchers’ feedback were analysed with NVivo12 using the six-step approach of theoretical thematic analysis (Kiger ME, Varpio L. 2020. *Medical Teacher* **42**:846–854), starting with familiarisation (full dataset), initial coding (identification of relevant excerpts in the full dataset) and a search for and review of the themes via a recursive process with regular discussions amongst the two members of the research team (EK, MK). Codes were defined and redefined based on discussions within the research team, leading to a set of 8 codes related to 3 main themes (see table). To triangulate the data on perceptions of the students, the three scientists were asked (by EK) to email their experiences. All three consented (their names are mentioned in the acknowledgements). Data were provided and analyzed in English for 2 of the renowned scientists and for 1 in Dutch.

**Table: Students’ learning experiences: main themes and subthemes**

| MAIN THEMES | SUBTHEMES |
| --- | --- |
| Affective experience  (including specific features) | - Useful and interesting - Enjoyable - Purpose - Format (time required, group format, discussions, ‘real life’) |
| Understanding the scientific process | - Being introduced to review-rebuttal process - Preparation for future job |
| Deepened understanding of course content | - Deep analysis and understanding - Designing new experiments |

**Figure: Distribution of quantitative students’ scores**


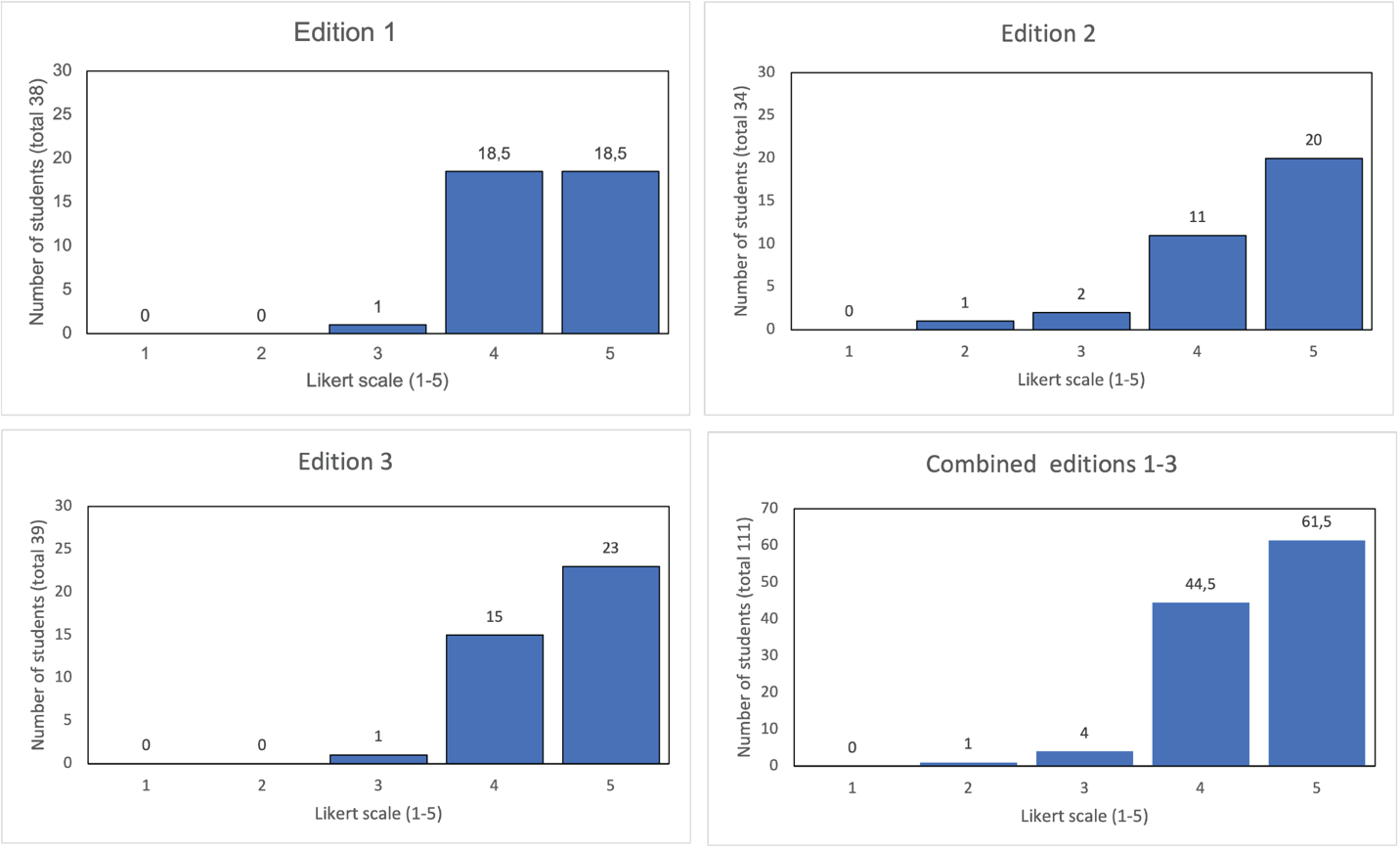

Supplement: Supplementary file 1. [file elife-102619-supp1.docx]
